# Investigation of cytokine imbalance in schizophrenia, assessment of the possible role of serum cytokine levels in predicting treatment response, prognosis and psychotic relapses

**DOI:** 10.1192/j.eurpsy.2025.618

**Published:** 2025-08-26

**Authors:** K. Bodnar, L. Hermán, R. Zsigmond, J. Réthelyi, Á. Dénes, K. Tóth

**Affiliations:** 1 Department of Psychhiatry and Psychotherapy, Semmelweis University; 2 Laboratory of Neuroimmunology, HUN-REN Institute of Experimental Medicine, Budapest, Hungary

## Abstract

**Introduction:**

Schizophrenia, a multisystem chronic psychiatric disorder of unknown etiology, is associated with several immune dysfunctions, including abnormal levels of circulating cytokines. Emerging data suggest a potential causative role for cytokines in schizophrenia symptom development. Furthermore, disease duration, symptom severity, aggressive behavior, and cognitive deficits are correlated with levels of inflammatory cytokines. Despite the development of new antipsychotics, the negative and cognitive symptoms of schizophrenia often do not respond adequately to pharmacotherapy.

**Objectives:**

1. Can there be a cytokine or cytokines among the different cytokine levels detected in schizophrenia that can be used as biomarkers of treatment response? 2. Can changes in cytokine levels indicate the occurrence of psychotic relapse? 3. Can changes in the cytokine level play a role in predicting the prognosis of the disease?

**Methods:**

We investigated cytokine levels in blood samples collected at hospital admission. Plasma levels of established inflammatory cytokines and chemokines have been measured by Cytometric Bead Array. The possible role of abnormal cytokine levels and their association with symptoms severity and their potential clinical implications have been investigated. The severity of the symptoms is monitored with the PANSS

**Results:**

16 schizophrenic patients who were hospitalized due to a psychotic relapse have been included. Blood samples were collected to measure cytokine levels, and the PANSS scale was recorded during a psychotic relapse. Additionally, we have included 11 age- and gender-matched healthy controls, from whom blood samples were also collected for cytokine measurement.. We found no differences plasma levels of G-CSF, GM-CSF, TNF, INF, VEGF, Il-6 and Il-10 in the patients compared with healthy controls. The levels of MCP-1 were higher in the schizophrenia patients than in the healthy control group.

**Conclusions:**

These data demonstrated elevated plasma levels of MCP-1 in schizophrenia patients. The role of MCP-1 in various CNS disorders that involve inflammation is emerging. Among these, chronic inflammation reportedly contributes to the onset and progression of neurodegenerative disorders such as Parkinson’s disease (PD), Alzheimer’s disease (AD), and Multiple Sclerosis. Links between circulating MCP-1 levels and the progression of schizophrenia has also been suggested by previous studies and further studies are required understand the underlying mechanisms.

**Significant outcomes:**

Patient with a psychotic relapse showed higher levels of plasma MCP-1 compared with healthy controls.

**Limitation:**

The number of samples is too low to detect a statistically significant differences in blood MCP-1 during a psychotic relapse. Involvement of additional patients is ongoing.

**Disclosure of Interest:**

None Declared

